# Measurement of Humanity Among Health Professionals: Development and Validation of the Medical Humanity Scale Using the Delphi Method

**DOI:** 10.2196/44241

**Published:** 2023-05-02

**Authors:** Jawdat Ataya, Issam Jamous, Mayssoon Dashash

**Affiliations:** 1 Medical Education Programme Syrian Virtual University Damascus Syrian Arab Republic; 2 Faculty of Dental Medicine Damascus University Damascus Syrian Arab Republic

**Keywords:** medical humanity, Medical Humanitarian Scale, scale, humanity, humanitarian, humane, Hippocratic oath, Delphi, development, patient centered, compassion, ethic, empathy, empathetic, validity, validation, person centered, the humanitarian aspect, students of medical colleges, Syria

## Abstract

**Background:**

Despite the importance of humanism in providing health care, there is a lack of valid and reliable tool for assessing humanity among health professionals.

**Objective:**

The aim of this study was to design a new humanism scale and to assess the validity of this scale in measuring humanism among Syrian health professional students.

**Methods:**

The Medical Humanity Scale (MHS) was designed. It consists of 27 items categorized into 7 human values including patient-oriented care, respect, empathy, ethics, altruism, and compassion. The scale was tested for internal consistency and reliability using Cronbach α and test-retest methods. The construct validity of the scale was also tested to assess the ability of the scale in differentiating between groups of health professional students with different levels of medical humanity. A 7-point Likert scale was adopted. The study included 300 participants including 97 medical, 78 dental, 82 pharmacy, and 43 preparatory-year students from Syrian universities. The Delphi method was used and factors analysis was performed. Bartlett test of sphericity and the Kaiser-Meyer-Olkin measure of sample adequacy were used. The number of components was extracted using principal component analysis.

**Results:**

The mean score of the MHS was 158.7 (SD 11.4). The MHS mean score of female participants was significantly higher than the mean score of male participants (159.59, SD 10.21 vs 155.48, SD 14.35; *P*=.008). The MHS mean score was significantly lower in dental students (154.12, SD 1.45; *P*=.005) than the mean scores of medical students (159.77, SD 1.02), pharmacy students (161.40, SD 1.05), and preparatory-year students (159.05, SD 1.94). However, no significant relationship was found between humanism and academic year (*P*=.32), university type (*P*=.34), marital status (*P*=.64), or financial situation (*P*=.16). The Kaiser-Meyer-Olkin test (0.730) and Bartlett test of sphericity (1201.611, *df*=351; *P*=.01) were performed. Factor analysis indicated that the proportion of variables between the first and second factors was greater than 10%, confirming that the scale was a single group. The Cronbach α for the overall scale was 0.735, indicating that the scale had acceptable reliability and validity.

**Conclusions:**

The results of this study suggest that the MHS is a reliable and valid tool for measuring humanity among health professional students and the development of patient-centered care.

## Introduction

The concept of medical humanity, as outlined in the Hippocratic Oath [1,2], emphasizes the ethical pledge among physicians to prioritize the well-being and autonomy of their patients [1]. Despite its importance in medical education and practice, there is a lack of valid and reliable methods for measuring humanity among medical student [3]. This is particularly important in the context of Syria, which has been affected by ongoing crisis, as it highlights the need for personnel with strong humanitarian values [4,5]. Therefore, this study was aimed at designing a scale and measuring humanism among health professional students.

The patient-centered perspective has been linked to humanity in health care [6]. Medical professionals are expected to respect patients’ autonomy, values, and aspirations; involve them in decision-making; and communicate effectively with them and their families [7]. Medical professionalism encompasses not only technical expertise but also ethical principles, patient-centered care, and humanistic characteristics [7]. To promote humanity in medical education, many medical schools have implemented instructional initiatives; however, these interventions have had limited success in promoting long-term humane care [8].

The majority of medical education professionals agree that practitioners should exhibit clinical abilities, expertise, attitudes, and behaviors toward patients [9,10]. However, there is a lack of suitable assessment instruments to evaluate and promote the concept of humanity in medical education [3].

Although several measures of the human aspect exist in health care, these measures are often limited in scope and mainly directed at nurses [11,12]. Furthermore, these measures adopt different methods than what was used in this study, such as focusing on specific dimensions of humanistic traits rather than a comprehensive assessment of the integration of values [13]. There is a need for a validated tool that comprehensively assesses the integration of core human values in health professional students and that is applicable across different health care professions [14,15].

This gap in the literature has led to inconsistent approaches in assessing medical humanity, which is essential for providing patient-centered care [15]. To address this gap, we designed and validated the Medical Humanity Scale (MHS) to assess the integration of 7 core human values in health professional students.

This study aimed to design a valid and reliable scale to measure humanity among medical, dental, and pharmacy students.

The study included the development of scale items; administration of the scale to a sample of medical, dental, and pharmacy students; and analysis of the scale’s psychometric properties. It was assumed that the scale would have good reliability and validity and be useful for evaluating and promoting humanity in medical education.

## Methods

### Design

This study included designing the MHS to assess the integration of 7 core human values in health professional students: respect, empathy, altruism, acceptance, consideration, appreciation, and compassion. Each of the 27 items in the scale evaluates multiple values simultaneously, providing a comprehensive assessment of the participant’s overall humanistic approach to patient care. The values are not treated as separate dimensions but rather as a unified block of humanistic traits.

The development process of the MHS involved several key steps to ensure its validity and reliability. The following frameworks were used:

1. Literature review: A comprehensive review of the existing literature on human values in health care was conducted to identify the core values to be included in the scale.
2. Expert involvement: A panel of experts in the field of medical education and health care was consulted to validate the items and ensure the content validity of the scale.
3. Pilot testing: A sample of health professional students was recruited to participate in a pilot test of the scale. The results were analyzed to assess the reliability and validity of the items and make any necessary revisions.
4. Reliability testing: The scale was tested for internal consistency and reliability using Cronbach α and test-retest methods.
5. Validity testing: The construct validity of the scale was tested by examining its ability to distinguish between groups of health professional students with different levels of medical humanity.
6. Refinement: Based on the results of the testing, the scale was further refined and finalized for use in research and assessment of medical humanity.

The variables that were taken into consideration when the items were created to reduce incorrect result interpretation were clarity, simplicity, and orientation. A 7-point Likert scale was adopted (1=Strongly Disagree, 2=Disagree, 3=Slightly Disagree, 4=Undecided, 5=Slightly Agree, 6= Agree, and 7=Strongly Agree) [16,17]. Ten of the 27 items were inverse, meaning the Likert-scale ratings would be inverted, whereas 17 of the 27 were positive. With at least one point for each item and a maximum of 7 points, the score ranges from 27 to 189 points.

Students from all medical faculties at the Faculty of Medicine, Dentistry, or Pharmacy were invited to participate in this study, and informed consent was obtained from all participants according to the Helsinki declaration [18].

Inclusion criteria included medical, dental, and pharmacy students from Syrian universities who were competent and cooperative to self-complete the web-based form using Google Form. For validity and reliability investigations, it is advised that the sample size in various statistical studies should be at least five to ten times the number of items on the scale [19]. This calculation yielded 270 individuals as the sample size for the 27-item scale. The sample size was increased to 300 participants to consider confounding factors.

Participants were asked to provide demographic information, including sex, academic year, university, social status, and economic status, in addition to answering the items of the MHS, which was used to collect the data.

### Instrument of Measurement: Delphi Method

In the beginning, the Delphi method was used to determine the optimal form of the scale to guarantee that all components measure the humanity aspect of all students.

### Statistical Analysis

Statistical analysis was performed using SPSS (version 25; IBM Corp). The mean, median, and SD of the total scores, together with all the descriptive data, were first analyzed. Additionally, the one-way ANOVA test and the 2-tailed *t* test were used to compare between groups and the total scores on the MHS at the significant level of .05. Cronbach α analysis and factors analysis were also performed. To gauge the efficacy of factor analysis, Bartlett test of sphericity and the Kaiser-Meyer-Olkin (KMO) measure of sample adequacy were used. The number of components was extracted using principal component analysis.

### Ethics Approval

The Syrian Virtual University’s ethics approval committee examined and authorized this study (number 287; date: April 18, 2022). All prospective participants provided their informed consent via an electronic registration form, during which they were made aware that the information they submitted would be kept private and used only for this research.

## Results

### Sample Characteristics

The sample included 300 students from Syrian medical faculties. The entire demographic statistics are shown in Table 1.

The total score statistics shown in Table 2.

Additionally, items underwent descriptive analysis. With at least 1 point and a maximum of 7 points for each question, the possible score ranges from 27 to 189 points. For each scale question, the mean, median, SD, and range were computed according to Table 3.

The highest scores were observed in question 21 (“I respect and keep all personal and medical secrets between me and patients”), where the average score of the responses was 6.85, according to Table 3. The lowest scores were found in inverted question 22 (“I make the choice on behalf of the helpless patient”), where the average score was 3.19.

**Table 1:** Demographic statistic.

Category		Participant (N=300), n (%)
**Sex**		
	Male	69 (23)
	Female	231 (77)
**Field of study**		
	Medicine	97 (32.3)
	Dentistry	78 (26)
	Pharmacy	82 (26)
	Preparatory year	43 (14.3)
**University type**		
	Private	32 (10.7)
	Public	268 (89.3)
**Marital status**		
	Single	290 (96.7)
	Married	9 (3)
	Divorced	1 (0.3)
**Employment status**		
	Employed	40 (13.3)
	Unemployed	260 (86.7)
**Financial situation**		
	Good	92 (30.7)
	Medium	192 (64)
	Bad	16 (5.3)

**Table 2:** Total score statistics of the Medical Humanity Scale.

Statistics	Value
Total score, mean (SD)	158.7 (11.4)
Total score, median	161
Skewness, mean (SD)	–.892 (.141)
Kurtosis, mean (SD)	1.205 (.281)

**Table 3:** Descriptive analysis of the Medical Humanity Scale items.

Items	Mean (SD)	Median	Mode	Range
1. I always strive to provide the best possible medical care to the patient by searching for and comparing the best treatment options for the patient.	6.72 (.567)	7	7	4-7
2. The patient’s needs are my priorities, so I take great care to listen to the patient and know his desires and interests.	6.47 (.803)	7	7	2-7
3. I can work overtime to provide the best patient service even if it is free of charge.	5.47 (1.389)	6	6	1-7
4. I treat all patients with kindness and always keep eye contact with them.	6.66 (.583)	7	7	4-7
5. I share treatment plans with the patient, encourage the patient to ask questions, and listen to their wishes for treatment options.	6.37 (.876)	7	7	1-7
6. Taking care of the patients leaves me exhausted.	3.75 (1.713)	3	3	1-7
7. I take care of the patient’s personal matters, as they are relevant to medical treatment.	5.16 (1.462)	5	6	1-7
8. I support euthanasia - when an incurable patient comes, I can help them die easily.	5.91 (1.706)	7	7	1-7
9. I support scheduling a time to visit remote and rural areas to treat patients even of it is free of charge.	5.99 (1.403)	7	7	1-7
10. I support treating low-income and needy people at a reduced or free cost.	6.59 (.769)	7	7	2-7
11. I respect all types of patients, regardless of their educational or living level, or even from which region they are.	6.88 (.364)	7	7	5-7
12. I can treat my enemy if he needs necessary medical help	6.21 (1.226)	7	7	1-7
13. I can treat people who disagree with me in opinion, principles and beliefs	6.74 (.618)	7	7	3-7
14. I may give false medical information in order to reduce the impact of the real news on the patient	4.82 (1.789)	5	7	1-7
15. I don't care about the patient’s gender, nationality, colour or race	6.90 (.367)	7	7	4-7
16. The patient’s financial level does not interfere with the type of medically necessary treatment for him	5.49 (1.791)	6	7	1-7
17. I support testing a new drug in rural or remote areas	6.11 (1.453)	7	7	1-7
18. I refuse to treat patients again if they ask for a medical opinion from another specialist	5.74 (1.581)	6	7	1-7
19. I complain to a medically or ethically disreputable doctor after I make sure of it	4.49 (1.679)	4	4	1-7
20. I benefit from poor and needy patients to conduct my scientific experiments on them	6.70 (.828)	7	7	1-7
21. I respect and keep all personal and medical secrets between me and patients	6.85 (.505)	7	7	2-7
22. I make the decision on behalf of the helpless patient	3.19 (1.658)	3	2	1-7
23. The quality of my medical treatment, if it is in a dispensary or a government hospital, differs from a private centre or hospital	5.97 (1.615)	7	7	1-7
24. I deeply sympathize with patients who have severe and acute condition	6.40 (.907)	7	7	2-7
25. I can’t sympathize with patients with sexually transmitted diseases	5.11 (1.687)	6	7	1-7
26. I support the treatment of patients with infectious diseases	5.76 (1.342)	6	7	1-7
27. People with special needs are not a priority for me	6.19 (1.316)	7	7	1-7

The MHS mean score differed significantly between male and female participants. Female students’ humanity scores (mean 159.59, SD 10.21) were significantly higher than male students’ scores (mean 155.48, SD 14.35; *P*=.008).

Furthermore, a significant difference between dental students and other majors was discovered. The mean score of MHS was significantly lower in dental students (154.12, SD 12.8; *P*=.005) than those of medical students (159.77, SD 10.09), pharmacy students (161.40, SD 9.52), and preparatory-year students (159.05, SD 12.73).

However, the *t* test and one-way ANOVA tests revealed no significant relationship between humanism and academic year (*P*=.32), university type (*P*=.34), marital status (*P*=.64), or financial situation (*P*=.16).

### Validity Analysis

#### Factor Analysis

The KMO and Bartlett tests were performed: KMO=0.730>0.7, which is near 1, and Bartlett test of sphericity=1201.611 (*df*=351; *P*=.01).

Table 4 presents the overall findings of the principal component analysis for the 27 items of the MHS. It displays the 9 extracted components that were kept, the initial eigenvalues, the variance percentages, and the cumulative percentages. A total variance before rotation accounted for 56.96% of the factors, each of which had an eigenvalue larger than one.

Since the percentage of variables between the first and second factors seems to be larger than 10% in Table 4, it confirmed that the scale only has one group.

**Table 4:** Initial eigenvalues findings of factor analysis.

Component	Total	Variance percentage (%)	Cumulative percentage (%)
1	4.652	17.231	17.231
2	1.866	6.911	24.142
3	1.562	5.784	29.926
4	1.444	5.348	35.274
5	1.369	5.069	40.343
6	1.194	4.424	44.767
7	1.161	4.300	49.066
8	1.123	4.161	53.227
9	1.009	3.737	56.964

#### Internal Consistency of the Scale: Pearson Correlation Coefficient

The internal consistency of the questions was measured by calculating the correlation coefficients between each question with the total score of its elements at the significance level of .01. The Pearson test was used and the results are shown in Table 5. It was found that the items were associated with the total score at the significance level (.01).

**Table 5:** The item-total correlations for each factor.

Item	Pearson correlation	*P* value (2-tailed)
1	0.311	<.001
2	0.403	<.001
3	0.510	<.001
4	0.365	<.001
5	0.328	<.001
6	0.392	<.001
7	0.305	<.001
8	0.294	<.001
9	0.481	<.001
10	0.458	<.001
11	0.358	<.001
12	0.471	<.001
13	0.385	<.001
14	0.227	<.001
15	0.287	<.001
16	0.372	<.001
17	0.312	<.001
18	0.317	<.001
19	0.157	.006
20	0.431	<.001
21	0.343	<.001
22	0.280	<.001
23	0.446	.001
24	0.342	<.001
25	0.330	<.001
26	0.384	<.001
27	0.469	<.001

### Reliability Analysis

#### Delphi Method

The validity and reliability of the scale were improved using the Delphi method [20]. The scale had 30 elements in it at first. Three experts in medical education, medical ethics, and humanity aspects were given the items to help them decide which were the best and most likely to accomplish the purpose and object of the humane scale. There were 2 rounds of reviews. Three elements were removed because they were redundant with other items in their meaning. Two additional things had their order reversed. Two more items were rephrased. The experts then confirmed the 27 items.

#### Cronbach α Internal Consistency Coefficient

The reliability of the scale was evaluated using the Cronbach α technique, and it was discovered that the scale items’ reliability coefficients were acceptable (0.735>0.700).

#### Spearman-Brown Coefficient

The average of the odd and even items was shown to be correlated using the Spearman correlation coefficient. The Spearman-Brown coefficient was used to adjust the correlation coefficients after that. Its result was 0.792>0.5, and the Guttman test had a value of 0.787, demonstrating its reliability.

## Discussion

### Principal Findings

Humanity is the cornerstone of medicine [7]. Medical care that values integrity, kindness, compassion, altruism, and respect for patients and their families can improve treatment outcomes and foster trust [7]. Studies have shown that doctors who are more compassionate have higher job satisfaction, lower rates of malpractice suits, and fewer process errors [21]. Humanistic medical care is also more likely to encourage patients to follow medical recommendations [22]

Medical professionals must possess not only medical knowledge and skills but also an ethical and positive outlook, as well as a strong connection with patients and their families [22]. Doctors should take into account the physical, emotional, and mental well-being of patients when providing care [23]. The development of a medical professional’s personality, including cognitive, moral, and behavioral traits, starts from their medical school [8,22,24]. Although scales measuring the humanistic aspect of medical professionals exist, they are mainly directed toward nurses and lack comprehensive assessment for other health professional students [4,11,13,15,25,26]. The number of standards for students at medical colleges is very limited, limited to different methods, and not comprehensive [13,15,26]. There is no Syrian scale that includes the human aspect of medical college students. Some scales were built by Syrian researchers to assess sympathy only among medical staff [4], which prompted the need to build a scale that includes a number of human values to measure the humanitarian aspect of medical college students.

This study was conducted to fill the gap by creating the MHS to measure the humanity aspect of health professional students, including integrity, respect, compassion, empathy, patient-centered care, giving, and altruism. A 7-point Likert scale was selected based on a study by Korkut Altuna and Arslan [17], which found no difference between 5-point and 7-point scales, and on previous studies that used a 7-point Likert scale [25]. The questionnaire was delivered electronically to students from various colleges and universities, including programs for medicine, dentistry, and pharmacy, using Google Forms. The scale uses a Likert scale, with 10 of the items being inverse and 17 being positive. A higher score would therefore indicate a more positive evaluation of the human values being assessed.

Based on previous work, content validity was assessed after designing the scale by evaluating the item legibility and content evaluation by 2 judges (MD and IJ) who are experts in medical education and supervisors of this study. In addition, the content validity was assessed by asking the opinions of participants and collecting their feedback quantitatively [27]. Furthermore, taking into consideration that the construct validity should be assessed, the authors were keen to assess whether the scores of the MHS are consistent with the hypotheses that the MHS should measure the intended construct. However, there was no similar instrument that can be used to assess the relationship of its scores with the scores of our instrument [28]. To establish the validity criterion of the MHS scale, we increased the sample size [29]. However, future work should apply the MHS to assess humanity among health professionals and investigate the overall correlation between its score and a measure of their humanity. For instance, an investigation can be undertaken to test how likely the test scores can predict future humanity.

The results showed that female students scored higher on the overall measure of humanity compared to male students [30]. However, the sample distribution was not equal between the two sexes, which may have affected the results. The study contradicts the findings of Fothan et al [30], who found no sex differences using the Patient-Practitioner Orientation Scale, but supports the findings of Roh et al [31], who found that female students are more empathic than male students using the Korean version of the Jefferson Scale of Physician Empathy. Dentistry students scored lower than students in medicine and pharmacy and first-year students. The percentage was lower for fifth-year and postgraduate students, and those at private universities scored higher than those at public universities. However, it is impossible to draw meaningful comparisons between these factors due to the demographic characteristics of the sample, such as a lack of married, divorced, or widowed students and those who did not work outside of school. The results showed that the financial status of medical college students did not influence their level of humanity.

### Limitations

The sample size used in this study is relatively small as it serves as an initial investigation that emphasizes the importance of assessing humanity among health professionals. Future work will further consider increasing the sample to ascertain our findings.

### Conclusion

The results of this study suggest that the MHS is a reliable and valid tool for measuring humanity among health professional students and the development of patient-centered care.

## Abbreviations

KMO: Kaiser-Meyer-Olkin
MHS: Medical Humanity Scale

## Data Availability

The data sets from which the results of this study were derived can be obtained from the corresponding author upon reasonable request.
